# Evaluation of NDEL1 oligopeptidase activity in blood and brain in an animal model of schizophrenia: effects of psychostimulants and antipsychotics

**DOI:** 10.1038/s41598-020-75616-2

**Published:** 2020-10-28

**Authors:** João V. Nani, Richard S. Lee, Camila M. Yonamine, Osvaldo A. Sant’Anna, Maria A. Juliano, Ary Gadelha, Jair J. Mari, Mirian A. F. Hayashi

**Affiliations:** 1 Departamento de Farmacologia, Escola Paulista de Medicina (EPM), Universidade Federal de São Paulo (UNIFESP), Rua 3 de maio 100, Ed. INFAR, 3rd floor, São Paulo, CEP 04044-020 Brazil; 2 Department of Psychiatry, Escola Paulista de Medicina (EPM), Universidade Federal de São Paulo (UNIFESP), São Paulo, Brazil; 3 National Institute for Translational Medicine (INCT-TM, CNPq/FAPESP/CAPES), Ribeirão Preto, Brazil; 4 Department of Psychiatry and Behavioral Sciences, Johns Hopkins University, Baltimore, MD USA; 5 Laboratory of Immunochemistry, Instituto Butantan, São Paulo, Brazil; 6 Department of Biophysics, Escola Paulista de Medicina (EPM), Universidade Federal de São Paulo (UNIFESP), São Paulo, Brazil

**Keywords:** Enzymes, Biochemistry, Neuroscience, Diseases of the nervous system, Schizophrenia

## Abstract

Nuclear distribution element-like 1 (NDEL1) enzyme activity is important for neuritogenesis, neuronal migration, and neurodevelopment. We reported previously lower NDEL1 enzyme activity in blood of treated first episode psychosis and chronic schizophrenia (SCZ) compared to healthy control subjects, with even lower activity in treatment resistant chronic SCZ patients, implicating NDEL1 activity in SCZ. Herein, higher NDEL1 activity was observed in the blood and several brain regions of a validated animal model for SCZ at baseline. In addition, long-term treatment with typical or atypical antipsychotics, under conditions in which SCZ-like phenotypes were reported to be reversed in this animal model for SCZ, showed a significant NDEL1 activity reduction in blood and brain regions which is in line with clinical data. Importantly, these results support measuring NDEL1 enzyme activity in the peripheral blood to predict changes in NDEL1 activity in the CNS. Also, acute administration of psychostimulants, at levels reported to induce SCZ-like phenotype in normal rat strains, increased NDEL1 enzyme activity in blood. Therefore, alterations in NDEL1 activity after treatment with antipsychotics or psychostimulants may suggest a possible modulation of NDEL1 activity secondary to neurotransmission homeostasis and provide new insights into the role of NDEL1 in SCZ pathophysiology.

## Introduction

Schizophrenia (SCZ) is a multifactorial mental disorder determined by both genetic and environmental factors. Nuclear distribution element-like 1 (NDEL1) oligopeptidase is the most prominent ligand amongst the binding partners of the protein product of *Disrupted-in-Schizophrenia 1* (*DISC1*) gene^1–3^. *DISC1* gene was first identified in a study conducted in a Scottish family with high prevalence of mental disorders, where its truncation t(1; 11)(q42;q14.3) was associated with SCZ as a potential genetic risk factor^4,5^. To date, the DISC1 protein is more likely recognized to be involved in a wide range of neuropathological conditions, and other DISC1-related biological functions, such as its potential involvement in dopaminergic signaling, motivated several studies aiming to understand its critical roles in molecular pathways involved in SCZ etiology and pathophysiology^6–8^. Interestingly, the NDEL1 and DISC1 protein complex formation was demonstrated to play essential roles in neurite outgrowth and neuron migration during embryogenesis, and disruption of DISC1/NDEL1 complex was suggested to affect the formation of specific brain structures^1,9–13^, with potential consequences for SCZ vulnerability^3,14^. We have demonstrated that the interaction with DISC1 competitively inhibits NDEL1 enzyme activity^1^, and beyond its interaction with DISC1, suppression of NDEL1 oligopeptidase activity was demonstrated to decrease neurite outgrowth^1,2^. In vitro studies suggested NDEL1 can hydrolyze small oligopeptides such as bradykinin (BK) and neurotensin (NT)^15–18^. BK has been proposed to augment neurogenesis, neuromodulation, and neuroprotection^19^, whereas NT was demonstrated to play an essential role downstream of typical antipsychotics such as haloperidol^20^. In addition, NT was also proposed to act as a natural endogenous antipsychotic^21^, and its mechanism of action could be mediated not only by dopamine but also by the serotonin signaling^22,23^. In other words, NDEL1 enzyme activity and its neuropeptide substrates, namely NT and BK, were independently associated with processes involved in SCZ pathophysiology and antipsychotic pharmacotherapy. However, it is still unclear whether alterations in NDEL1 enzyme activity are able to modify the levels of any neuropeptides in vivo, as technical limitations have hindered past efforts^25^. More recently, we have also demonstrated that NDEL1 knockout *C. elegans* had an important imbalance in the serotonin pathway with significant deficits in their response to typical antipsychotic haloperidol compared to the atypical antipsychotic clozapine, reinforcing the impaired serotonin pathway with NDEL1 expression^24^. Together, these lines of evidence supported evaluating the involvement of NDEL1 enzyme activity in SCZ.

Previously, we have demonstrated in clinical samples a significantly lower NDEL1 activity in the plasma of chronic SCZ patients compared to age- and sex-matched healthy controls (HCs)^26^. In this same chronic SCZ cohort, we have also found significantly lower NDEL1 enzyme activity in treatment-resistant SCZ (TRS) patients, with TRS being defined by the International Psychopharmacological Criteria (IPAP) as a failure to respond to 4–6 weeks of monotherapy with two different antipsychotics in adequate dose [www.ipap.org] and no significant association to any specific clinical data or to the antipsychotics used^26^. Interestingly, the drug of choice for treating TRS patients is the atypical antipsychotic clozapine (which also acts on serotonin pathways), as these patients usually are not responsive to treatment with typical antipsychotics (which mainly act on dopamine pathways)^27,28^. Studies have indicated that TRS may be particularly linked to aberrant neurodevelopment, as impaired cortical and striatal functional connectivity was observed in TRS^28–30^. In addition, we have also reported significant differences in NDEL1 enzyme activity between antipsychotic naïve first-episode psychosis (FEP) patients compared to HCs after treatment with risperidone, with a progressive decrease in NDEL1 activity observed during 2 months and/or 1 year of treatment with (atypical antipsychotic) risperidone, during which a significant improvement of symptoms was observed in all patients, with significant positive correlation observed between NDEL1 activity decrease and these symptoms amelioration, as assessed by PANSS^31^. Finally, lower NDEL1 activity was also observed in medicated euthymic individuals with bipolar disorder type 1 compare to HCs^32^. These studies implicate NDEL1 activity in SCZ and, now, necessitate the use of in vivo models to delineate the role of NDEL1 in the potential effects of these antipsychotics on NDEL1 enzyme activity.

The spontaneously hypertensive rat (SHR) strain was first characterized as an animal model to study hypertension due to its inherent high blood pressure^33^. This rat strain was later proposed as an animal model for attention deficit hyperactivity disorder (ADHD)^34^, and more recently, it was also recognized as an animal model for studying SCZ due to the presence of several behaviors that mimic the symptomatology observed in SCZ patients. Of the several behavioral deficits observed in SHR strain, reduced PPI (prepulse inhibition of startle), which denotes a deficit in sensorimotor processing that is linked to the inability of SCZ patients to filter out extraneous information, is the most interesting^35^, as this PPI deficit of SHRs was shown to be potentiated by psychostimulants and reversed by antipsychotic medications^36^. Behaviors mimicking positive and negative symptoms, such as hyperlocomotion and reduction in social interaction, respectively, were also noticed in SHRs compared to their normotensive counterpart strain Wistar rats (NWRs), and these deficits were also ameliorated by typical or atypical antipsychotic treatment^37,38^. Moreover, administration of the psychostimulant amphetamine also exacerbated SCZ-like behaviors in these animals^37^, which also allowed us to demonstrate that these behavioral changes were not related to high blood pressure^39^.

First, we sought to further investigate the potential of NDEL1 enzyme activity as a clinical biomarker of SCZ^26^. Now, to better understand its role in the neurobiology of SCZ, by strictly implementing the same regimen and protocols previously shown to reverse the SCZ-like behavioral dysfunction in the SHR rat^36–38^, we pursue the following objectives: (1) compare baseline blood NDEL1 enzyme activity levels between SHRs and the control NWRs; (2) compare blood NDEL1 enzyme activity levels before and after treatment with typical and atypical antipsychotics in both rat strains; (3) compare baseline brain NDEL1 enzyme activity levels between the SHRs and NWRs; (4) compare brain NDEL1 enzyme activity levels before and after treatment with typical and atypical antipsychotics in both rat strains; and (5) compare blood NDEL1 enzyme activity levels after the administration of psychostimulants. To the best of our knowledge, this study is the first to concomitantly measure NDEL1 enzyme activity in blood and brain of an animal model for SCZ, before and after treatment with typical and atypical antipsychotics, and to evaluate the in vivo effect of these antipsychotic drugs and psychostimulants on NDEL1 enzyme activity. The main aim of this work is to compare the effect of these drugs with the previously published clinical data with antipsychotic-naïve FEP individuals, before and after treatment with risperidone^31^, and with chronic treated SCZ patients relative to HCs^26^.

## Material and methods

### Animals

Three to five months-old male normotensive Wistar rats (NWRs) and spontaneously hypertensive rats (SHRs) from our colony were housed under controlled conditions, such as temperature (22–23 °C) and lighting (12/12 h light/dark cycle, lights on at 07:00 AM). Groups of 4 animals were kept in Plexiglas cages (41 × 34 × 16.5 cm) with free access to food and water. The animals were maintained in accordance with the guidelines of the Committee on Care and Use of Laboratory Animal Resources, National Research Council, USA. This study was approved by the Ethical Committee of the Universidade Federal de São Paulo (UNIFESP/EPM), under CEUA Nº 7290170315. All rats used for each experiment were drug-naive.

### Drugs

Haloperidol (Sigma-Aldrich, St. Louis, MO, USA), clozapine (Pinazan, Laboratório Cristália, São Paulo, Brazil), amphetamine (AMPH—Sigma-Aldrich, St Louis, USA) and lisdexanfetamine dimesylate (LDX—Vynvase, Shire LLC, São Paulo, Brazil) were dissolved in saline. All drug and vehicle solutions were injected by intraperitoneal (*ip*) route in a volume of 1 mL/kg of body weight.

### Treatment with antipsychotics

For the treatments with antipsychotics, 4 month-old male animals from NWR and SHR strains were housed by strain and kept for a month to acclimate before starting the daily treatment for 30 consecutive days in which: group I (control) received vehicle (saline 0.9%, 1 mL/kg), group II were treated with haloperidol (0.5 mg/kg, *ip*), and group III were treated with clozapine (2.5 mg/kg, *ip*).

### NDEL1 enzyme activity measurements in rat plasma after the treatment with psychostimulants

Male NWR animals (4 months old) were used to compose the following groups: group I (control)—3 animals received the vehicle (saline 0.9%), group II (AMPH)—3 animals received a single dose of AMPH (0.5 mg/kg), and group III (LDX)—3 animals received a single dose of LDX (0.5 mg/kg). NWR treated with psychostimulants or receiving saline had their blood collected 2 h after the injection. Blood was collected by caudal punction and then delivered into tubes containing heparin.

### Animal sample preparation

#### Blood collection and serum preparation

Blood of NWR and SHR animals (untreated, receiving vehicle saline or treated with antipsychotics for one month) was collected individually, in microcentrifuge tubes without anticoagulant following euthanasia by decapitation. After blood was allowed to clot at room temperature, serum samples were recovered after centrifugation at 1000—2000 × g for 10 min at 4 °C. Aliquots of serum were stored in microcentrifuge tubes at − 20 °C until use, as previously described^31,39^. NWR treated with psychostimulants or receiving saline had their blood collected into tubes containing heparin, and after centrifugation, plasma was carefully aliquoted into labeled clean tubes, before storage at – 20 °C until use.

#### Brain dissection and preparation of tissue homogenates

Specific brain regions were dissected using the bregma distance for each region as follow: prefrontal cortex (+ 5.1 mm; + 2.7 mm), hippocampus (− 2.0 mm; − 4.8 mm), and striatum/nucleus accumbens (+ 2.1 mm; + 0.2 mm). Dissected tissues were immediately frozen and kept at – 80 °C until use. Brain tissues were carefully defrosted on wet ice before homogenization in 50 mM HEPES, pH 7.9, with 500 mM NaCl buffer (4 mL/g of tissue weight), for 3 times with 30 s per cycle, with 1 min of interval between each cycle, immersed in a wet ice bath using a Polytron homogenizer (Fisher Scientific, Pittsburgh, PA, USA). The homogenates were then centrifuged at 20,000 × g for 10 min at 4 °C, and the supernatant was recovered and used for the NDEL1 enzyme activity measurements. Protein concentration of these samples was determined (at 595 nm) by the Bradford method (Bradford Protein Assay, BioRad, Hercules, CA, USA).

### NDEL1 enzyme activity measurements

NDEL1 enzyme activity was measured as previously described by our group^23^. Briefly, the hydrolysis of substrate was monitored at 37 °C by measuring the fluorescence (λ_Ex_ = 420 nm and λ_Em_ = 320 nm) in a 96-well black plate using a F-7000 spectrofluorimeter (Hitachi Ltd., Ibaraki, Japan). For each well, we added: 10 μL of serum or 2–4 μL of tissue homogenate and 200 μL of buffer (NaCl 100 mM, 50 mM Tris–HCl, pH 7.4) containing 5 μM of fluorescence resonance energy transfer (FRET) substrate (Abz-GFSPFRQ-EDDnp), either in the absence or presence of 10 μL of heat-inactivated NDEL1 antibody (NO_AB_ inhibitor). This polyclonal antibody shows specific inhibitory activity against NDEL1^1,13,17,18^, and the NDEL1 specific activity was defined here as the rate of hydrolysis in the absence of NO_AB_ inhibitor minus the rate measured in the presence of this specific antibody, as previously described^1^.

Arbitrary Units of Fluorescence (AUF) values were obtained by angular coefficient of each reaction, in which 1 μM of substrate corresponds to 2,870 AUF/s (per second) using the F-7000 fluorimeter. The NDEL1 activity was calculated using the formula: μM/min = AUF/s × 60 for serum and μM/min/mg = AUF/s × 60/mg of total protein added to the reaction for tissue homogenates.

### Blood pressure measurements

The measure of the systolic blood pressure (BP) was measured as previously described by our group^39^. Briefly, BP of rats treated with psychostimulants was measured by a non-invasive pressure system MRBP system (IITC Life Science, California, USA), which measures diastolic BP at the tail of rat. Animal were preconditioned in the system (3–4 times before the experimental procedure), in order to avoid the increase of BP eventually caused by stress, and maintained in a temperature between 35–37 °C that allowed the dilation on the caudal artery, promoting higher accuracy and precision in the measurements of the BP.

### Data analysis

Standard parametric (Student’s *t* test and two-way ANOVA) and non-parametric (Spearman’s Rho) tests were applied accordingly to variables type and distribution. All distribution was checked using a Kolmogorov-Smirnoff test. All results are expressed as the value of mean ± standard deviation. Data analyses were performed using the GraphPad Prism version 7.00 for Windows (GraphPad Software, La Jolla California USA). Significance threshold was considered at *p* < 0.05.

## Results

### NDEL1 enzyme activity in the serum of drug-naïve animals

The mean values for NDEL1 enzyme activity in the serum of 5 months-old drug-naïve normotensive Wistar rats (NWR) and spontaneously hypertensive rats (SHRs) were determined as 4.2 ± 0.7 and 7.1 ± 1.3 nM/min, respectively, showing a significantly higher NDEL1 activity in the blood of untreated SHR compared to NWR animals (Student’s *t* test (t = 3.09, df = 15, *p* = 0.014) (Supplemental Fig. 1).

**Figure 1:**
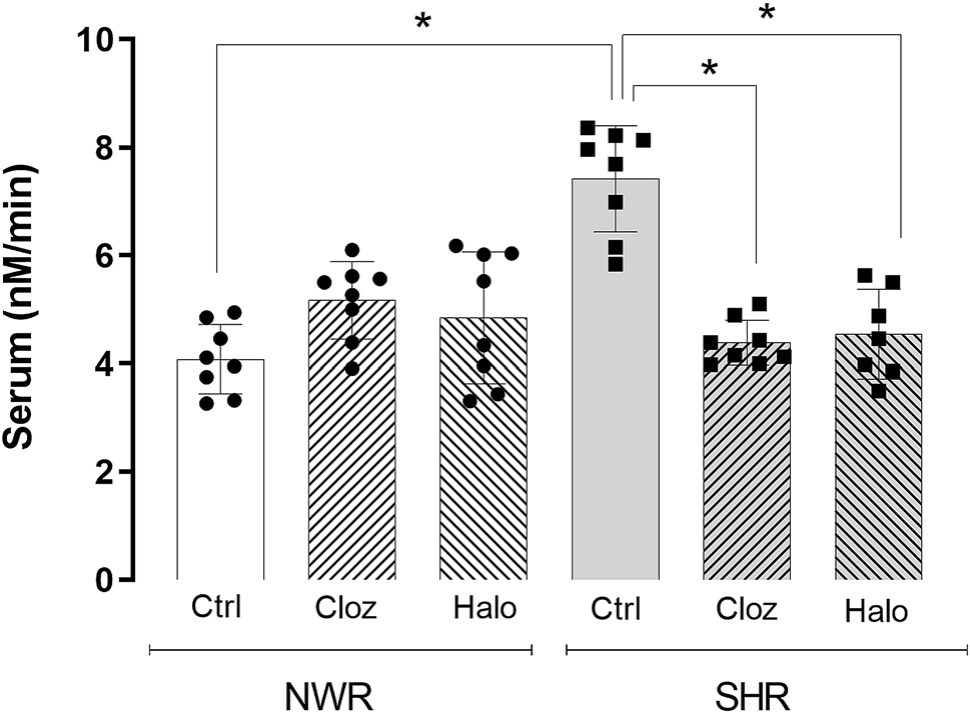
NDEL1 enzyme activity in the serum of normotensive Wistar rats (NWRs) and spontaneously hypertensive rats (SHRs), after treatment with typical or atypical antipsychotics. Five months old NWR and SHR animals were treated daily with the typical antipsychotic haloperidol (Halo, 0.5 mg/kg, *ip*), atypical antipsychotic clozapine (Cloz, 2.5 mg/kg, *ip*) or vehicle (saline, 1 mL/kg, *ip*), during 30 days. Blood was collected 24 h after the last administration of antipsychotics or vehicle, and the collected serum was used for the NDEL1 activity measurements (N = 8, two-way ANOVA, post-hoc test Tukey for multiple comparisons, *p < 0.05 compared to the control group).

### NDEL1 enzyme activity in the serum of typical or atypical antipsychotics treated animals

Treatment of 5 months-old adult rats, which were acclimated for a month before starting the daily treatment with typical (haloperidol) or atypical (clozapine) antipsychotics for 30 consecutive days, showed a significant decrease in NDEL1 enzyme activity only in the serum of SHR animals, whereas NDEL1 activity in the blood of NWR animals was not significantly affected compared to respective controls receiving saline (Fig. 1 and Table 1). A two-way ANOVA test showed a significant interaction with treatment only in the SHR strain (F (2, 42) = 8.68, *p* = 0.0017), and a significant correlation between NDEL1 enzyme activity and treatment was also observed only in the SHR strain (Spearman’s rho = − 0.617, *p* = 0.0250).

**Table 1:** NDEL1 enzyme activity (nM/min) in the serum of rats after 30 days treatment.

Treatment	NWR (N = 8)	SHR (N = 8)
Saline	4.1 ± 0.6	7.4 ± 1.2*
Clozapine	5.2 ± 0.7	4.4 ± 0.4^#^
Haloperidol	4.8 ± 1.3	4.5 ± 0.8^#^

^#^*p* < 0.05 for ANOVA compared to respective control animal treated with saline; *p < 0.05 for ANOVA comparing the SHR against the NWR under the same condition.

### NDEL1 enzyme activity in several brain regions of drug-naïve rats

**Figure 2:**
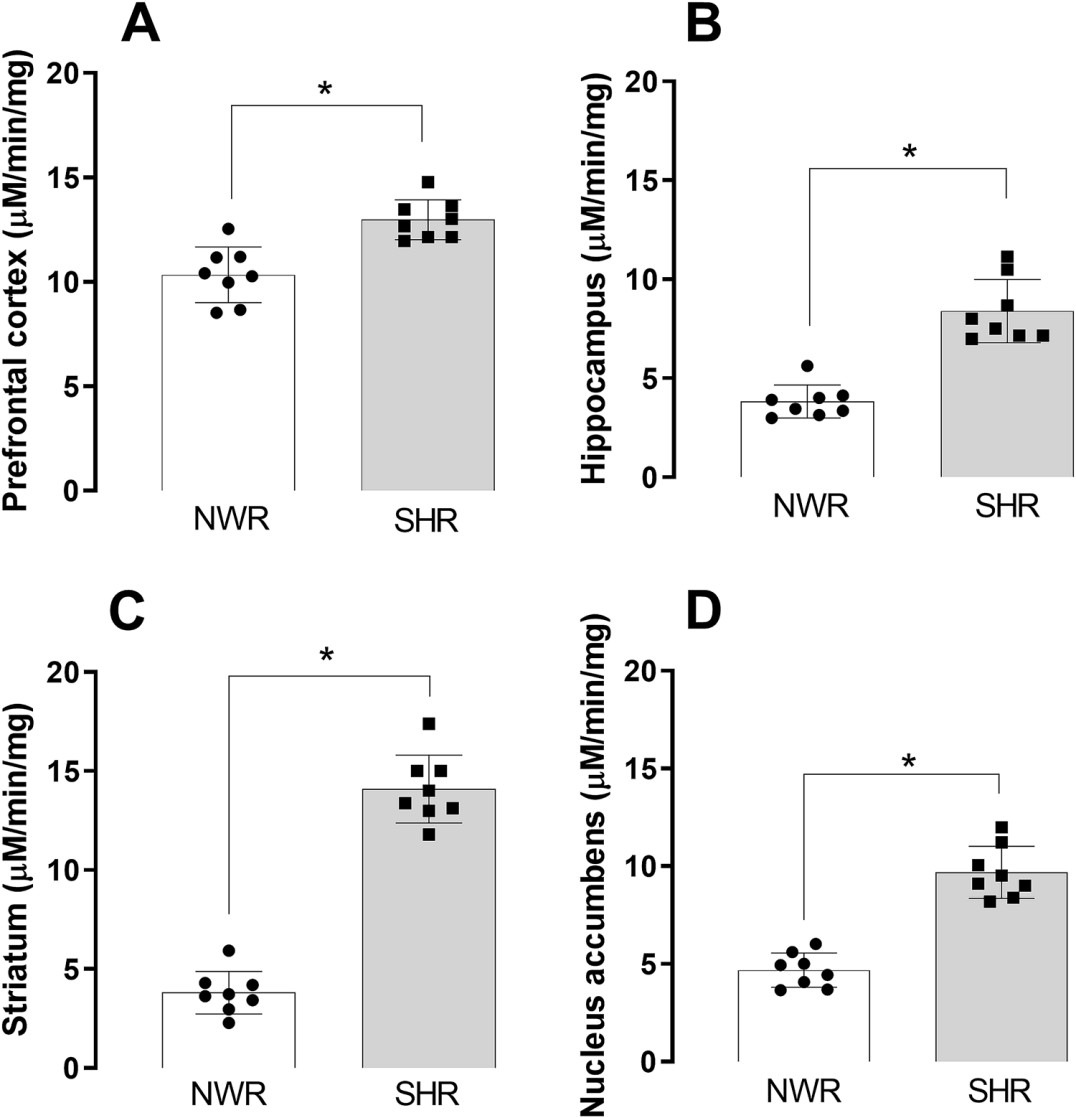
NDEL1 enzyme activity in selected brain regions of Drug-naïve normotensive Wistar rats (NWR) and spontaneously hypertensive rats (SHR) animals at baseline. Selected brain regions of 5 months old male rats were collected, and soluble fraction of brain tissue homogenate was used to measure the NDEL1 activity (N = 8, *p < 0.05, *t*-Student test, post-hoc Bonferroni test for multiple comparisons).

We compared baseline NDEL1 activity in the brain of NWR and SHR animals. In the prefrontal cortex, hippocampus, striatum and nucleus accumbens of drug-naïve NWR, the NDEL1 enzyme activity was 10.5 ± 1.3, 3.7 ± 0.8, 3.9 ± 1.0, and 4.7 ± 0.9 μM/min/mg of protein, respectively, while the values for the NDEL1 activity in the drug-naïve SHR were 12.9 ± 0.9, 8.2 ± 1.6, 14.0 ± 1.7, and 9.9 ± 1.3 μM/min/mg of protein, respectively (Fig. 2). Statistically significant, higher NDEL1 enzyme activity was observed in all four brain regions tested: prefrontal cortex (t = 4.529, df = 14, *p* < 0.0020) hippocampus (t = 7.153, df = 14, *p* < 0.0004), striatum (t = 14.38, df = 14, *p* < 0.0004) and nucleus accumbens (t = 8.877, df = 14, *p* < 0.0004) (Fig. 2).

### NDEL1 enzyme activity in several brain regions of typical or atypical antipsychotic-treated rats

**Figure 3:**
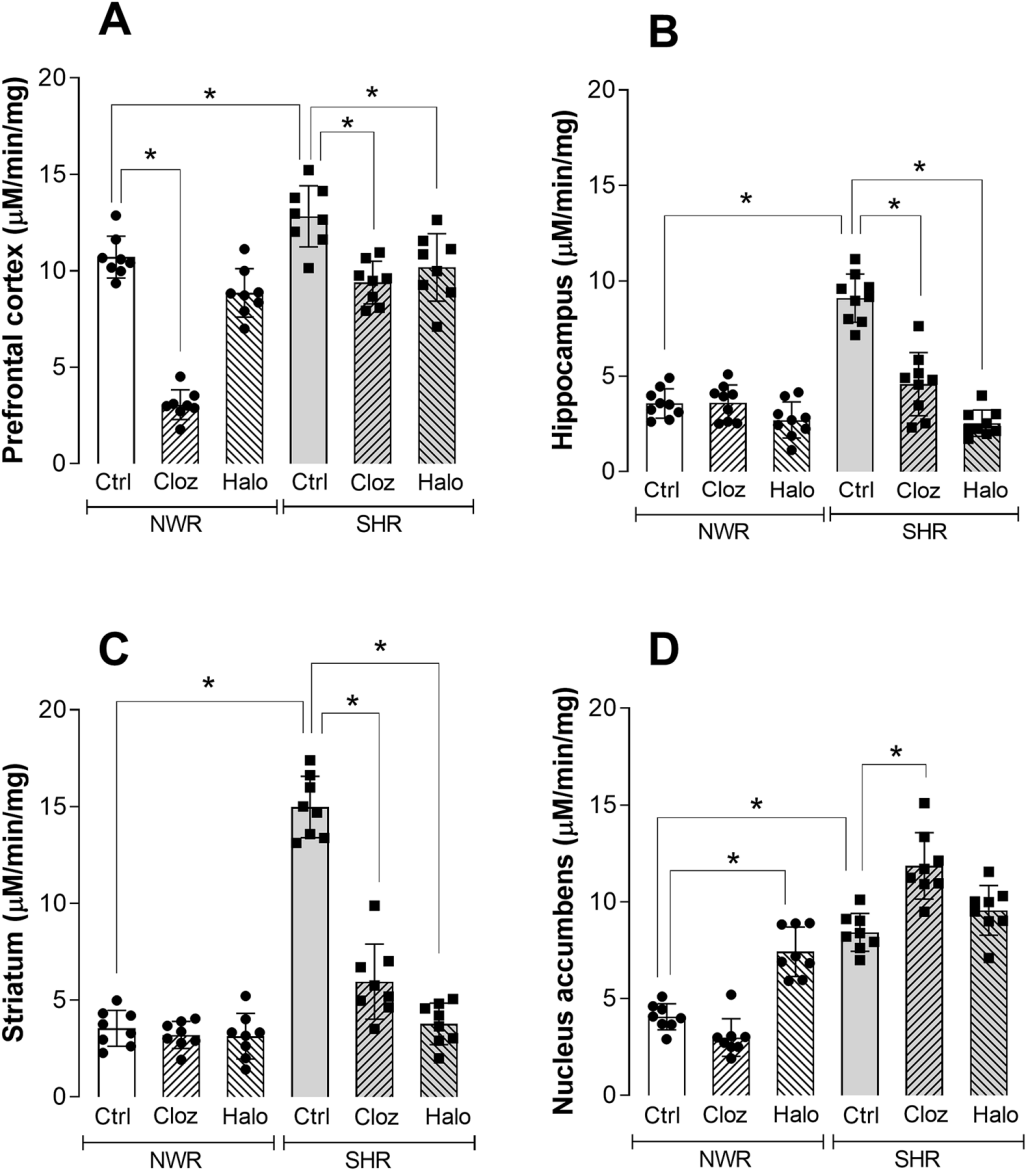
NDEL1 enzyme activity in selected regions of the brain of normotensive Wistar rats (NWRs) and spontaneously hypertensive rats (SHRs) after treatment with typical and atypical antipsychotics or receiving saline for 30 days. Selected brain regions of rats treated for 30 consecutive days with the typical (Halo) or atypical (Cloz) antipsychotics were collected after animal euthanasia, and soluble fraction of freshly prepared tissue homogenate was used to measure the NDEL1 enzyme activity (N = 8, *p < 0.05, two-way ANOVA, post-hoc test Tukey for multiple comparisons).

After treatment with typical or atypical antipsychotics, three of the four brain regions in the SHR strain (namely prefrontal cortex, hippocampus and striatum) showed a significant decrease in NDEL1 enzyme activity (Fig. 3A–C), similarly as observed for NDEL1 activity in the serum of these animals (Fig. 1 and Table 1). A two-way ANOVA test confirmed the significant effect of treatment for the prefrontal cortex (F (2, 42) = 13.45, *p* = 0.0001), hippocampus (F (2, 42) = 17.12, *p* = 0.0001) and striatum (F (2, 42) = 29.16, *p* = 0.0001) only in the SHR strain. In addition, a significant correlation was also observed between NDEL1 activity and the treated animal groups in the hippocampus (Spearman’s rho = − 0.940, *p* = 0.0010) and striatum (Spearman’s rho = − 0.926, *p* = 0.0010) of SHR strain.

In the prefrontal cortex (Fig. 3A), a significant decrease in NDEL1 activity was observed in the NWR strain for the treatment with clozapine (post-hoc Tukey test *p* = 0.0001), while for the SHR strain, NDEL1 activity was statistically significantly lower, after the treatment with either antipsychotic, as shown by a post-hoc Tukey test (*p* < 0.0001 for clozapine, and *p* < 0.0013 for haloperidol). In addition, a correlation for NDEL1 activity and treatment with either antipsychotic was statistically significant in prefrontal cortex in the SHR strain (Spearman’s rho = − 0.645, *p* = 0.0180).

Interestingly, in the nucleus accumbens, only treatment with haloperidol increased NDEL1 activity in the NWR animals (post hoc Tukey test *p* = 0.0151), while a significant increase in NDEL1 activity in SHR was observed only after treatment with clozapine (post-hoc Tukey test *p* = 0.0124), but not with haloperidol (post-hoc Tukey test *p* = 0.6514) (Fig. 3D). We also observed a significant main effect for treatment in this specific brain region (F (2, 42) = 17.62, *p* = 0.0010), with no significant correlation between treatment with antipsychotics and NDEL1 enzyme activity in the SHR strain (Spearman’s rho = 0.146, *p* = 0.6350).

It is also noteworthy that in the majority of brain regions analyzed in the NWR strain, NDEL1 enzyme activity was not significantly affected by treatment with either antipsychotic (typical or atypical) (Fig. 3 and Table 2), with the only exception for the decrease in NDEL1 enzyme activity in the prefrontal cortex following treatment with clozapine but not with haloperidol (Fig. 3A). Also, a significant increase in NDEL1 activity was observed in the nucleus accumbens of the NWR strain following treatment with haloperidol but not with clozapine (Fig. 3D).

**Table 2:** NDEL1 enzyme activity in brain regions after 30 days treatment (µM/min/mg).

Region	Treatment	NWR (N = 8)	SHR (N = 8)
Prefrontal cortex	Saline	10.7 ± 1.3	12.1 ± 1.6*
	Clozapine	3.0 ± 0.7^#^	9.3 ± 1.1^#^
	Haloperidol	8.8 ± 1.3	10.0 ± 1.8^#^
Hippocampus	Saline	3.5 ± 0.7	9.0 ± 1.3*
	Clozapine	3.6 ± 1.0	4.5 ± 1.7^#^
	Haloperidol	2.6 ± 0.9	2.5 ± 0.7^#^
Striatum	Saline	3.4 ± 1.1	14.7 ± 1.5*
	Clozapine	3.2 ± 0.7	6.1 ± 2.0^#^
	Haloperidol	3.1 ± 1.1	3.7 ± 1.1^#^
Nucleus accumbens	Saline	4.0 ± 0.6	8.5 ± 0.9*
	Clozapine	3.0 ± 0.9	12.0 ± 2.0^#^
	Haloperidol	7.2 ± 1.5^#^	9.8 ± 1.2

^#^*p* < 0.05 for ANOVA compared to respective control animal treated with saline; *p < 0.05 for ANOVA comparing the SHR against the NWR under the same condition.

### NDEL1 enzyme activity measurements in rat plasma after treatment with psychostimulants

**Figure 4:**
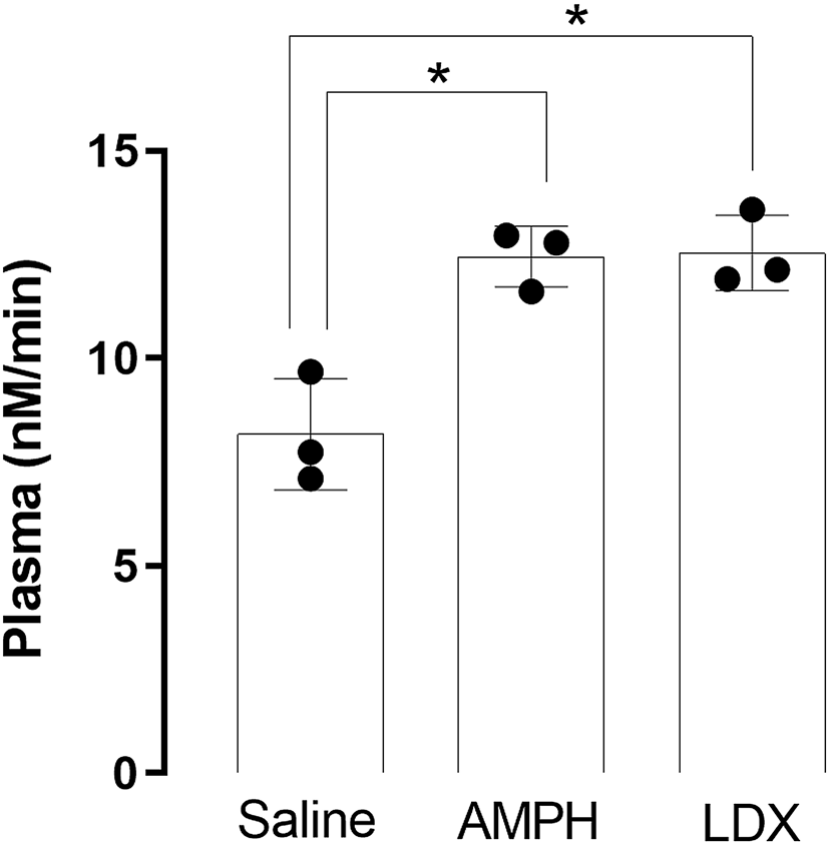
NDEL1 enzyme activity in the plasma of normotensive Wistar rats (NWR) after administration of psychostimulants. The animals received vehicle (saline), amphetamine (AMPH, 0.5 mg/kg, *ip*) or lisdexanfetamine (LDX, 0.5 mg/kg, *ip*), in a volume of 1 mL/kg of body weight. The NDEL1 enzyme activity was measured in the plasma obtained from the blood collected 2 h after administration of these psychostimulants or vehicle (N = 3, one-way ANOVA, *p < 0.05).

To evaluate the potential effects of dopamine release on NDEL1 enzymatic activity after the administration of psychostimulants, this activity was measured in plasma of animals showing significantly higher NDEL1 enzyme activity 2 h after single *ip* administration of either amphetamine (AMPH, 12.4 ± 0.7 nM/min, *p* = 0.0053) or lisdexanfetamine dimesylate (LDX, 12.5 ± 0.9 nM/min, *p* = 0.0048) compared with animals from the saline control group (8.1 ± 1.3 nM/min) (Fig. 4). In spite of the fact that significant increase in the animal blood pressure (BP) was observed only after the single administration of AMPH, while no significant difference was noticed for the LDX or vehicle groups (Table 3).

**Table 3:** Arterial blood pressure (BP, mmHg) of rats before and after the treatment with stimulants.

Treatment	NWR (N ≥ 3)	
	Before	After
Saline	146.6 ± 3.5	143.0 ± 3.6
Amphetamine (AMPH)	138.8 ± 3.0	160.3 ± 3.3*
Lisdexanphetamine (LDX)	135.4 ± 1.7	135.0 ± 3.8

*p < 0.05 for t-student test compared to the respective animal before treatment.

## Discussion

In the present study, a specific rat model for schizophrenia (SCZ) was used to evaluate the effects of a long-term treatment with typical or atypical antipsychotics on NDEL1 enzyme activity measured in the blood and selected brain regions of an animal model for SCZ and a control normal rat strain. This animal model, namely spontaneously hypertensive rat (SHR), was chosen because this specific strain exhibits several SCZ-like dysfunctions, such as impaired prepulse inhibition of startle (PPI) and contextual fear conditioning, which are phenotypically similar to deficits induced by acute amphetamine administration in normal Wistar rats (NWRs)^36^. PPI dysfunction is often reported in psychiatric disorders, including SCZ^40^, and it has been extensively used in translational operational measures of sensorimotor gating, since it can be evaluated in all mammals, including rats and humans^41,42^. The pathological mechanism underlying the positive and negative symptoms of SCZ patients may, at least in part, overlap with the SCZ-like behavioral phenotypes observed in SHR strain. Moreover, this PPI deficit of SHRs was shown to be reversed by antipsychotic medications^36^. Therefore, using these rat strains we have first evaluated the differences in NDEL1 activity at baseline, in blood (peripheral tissues) and several selected brain regions (CNS) of these animals. In addition, we have also compared the effects of typical and atypical antipsychotics on NDEL1 enzyme activity in both SHR and NWR strains. And finally, we also evaluated the effects of psychostimulants on NDEL1 enzyme activity.

We report here that the NDEL1 activity is higher in the blood of SHR compared to control NWR animals, and this difference was also observed in several brain regions of these animals at baseline. These findings are consistent with our clinical studies demonstrating increased *NDEL1* expression in the blood of antipsychotic-naïve first episode psychosis (FEP) SCZ patients^43^.

It is well-known that antipsychotics and/or neuroleptics improve the symptoms of SCZ patients mainly by acting on dopamine and/or serotonin pathways^44^. Treatment with either typical or atypical antipsychotics, under conditions previously described to reverse several behavioral deficits in the SHR strain^36–38^, led to a significant decrease in NDEL1 activity in the blood and brain of the SHR animals. Interestingly, treatment of FEP individuals with risperidone for 2 months or one year also showed significant decrease in NDEL1 activity^31^. In the same way, lower NDEL1 enzyme activity was observed in chronically treated SCZ patients compared to HCs matched by sex and age^26^, possibly supporting our hypothesis that NDEL1 activity correlates with the pharmacological effect of antipsychotics.

It is also worthwhile to emphasize that the NWR control animals, in contrast to SHR animals, did not show important changes in NDEL1 activity in the blood and majority of brain regions, even after long-lasting treatment with either typical or atypical antipsychotics, which is in line with studies reporting no changes in the behavior of control NWR animals^36^. Unfortunately, specific comparisons with clinical observations are limited in this regard, as administration of antipsychotics to healthy volunteers with no clinical indication is not ethically acceptable, and collecting fresh human brain samples from patients or healthy volunteers for measuring NDEL1 enzyme activity is not technically feasible. Accumulating evidences suggested that peripheral tissues, such as blood, can be used as surrogates for the central nervous system (CNS), although their validity has been questioned^45–47^. Herein, at least for NDEL1 enzyme activity, we could demonstrate the good correlation between the values measured in the peripheral blood and in several selected brain regions of 2 different rat strains, before (at baseline) or after a long-term treatment with typical or atypical antipsychotics, confirming the validity of measuring NDEL1 enzyme activity in the peripheral blood to predict changes of NDEL1 activity in the CNS in those clinical studies conducted by us previously^26,31,32^.

The nucleus accumbens has been implicated as an important location of dysfunctions in SCZ and antipsychotic drug action^48^. As part of the limbic circuitry, the hippocampus and prefrontal cortex were also analyzed here. In addition, deficits in neuronal network synchrony in these regions are also classically associated with SCZ^49–52^. Since, in addition to the prefrontal cortex, the striatum showed the highest expression of NDEL1 in the brain^17,53^, this brain region was also included in the present study. A significant decrease in NDEL1 activity was observed in the prefrontal cortex, hippocampus, and striatum of SHR animals, with no changes in NWR brain regions, after the treatment with either typical or atypical antipsychotics. Correlations between NDEL1 activity and treatment was statistically significant for SHR in the prefrontal cortex, hippocampus, and striatum, also confirming the validity of measuring NDEL1 enzyme activity in peripheral blood or CNS of this animal model of SCZ, aiming to understand the NDEL1 activity measures in peripheral blood of treated SCZ and/or FEP patients. Therefore, it is worth to highlight here that such correlation between NDEL1 activity and treatment was not valid in control NWR animals after long-term treatment with typical or atypical antipsychotics, although the correlation of NDEL1 activity levels between blood and brain was observed for both rat strains, namely NWR and SHR.

Moreover, an important increase in NDEL1 activity was also observed in the nucleus accumbens of SHR animals only after treatment with atypical antipsychotic clozapine. It is well-known that dopamine levels preferentially increase in this specific brain region only after treatment with clozapine, but not with the typical antipsychotic haloperidol^54^. Therefore, we suggest that these changes are more likely related to the chronic administration of these typical and atypical antipsychotics, as these medications were shown to lead to molecular neuroadaptive changes in the brain, depending on the type of antipsychotic used. For instance, clozapine has an effect on mesocorticolimbic neurotransmission (increasing dopamine and serotonin concentrations), whereas haloperidol alters glutamate and GABA neurotransmission^55^. Moreover, the strong correlation of serotonin pathway with NDEL1 was consistently demonstrated^24^. These alterations potentially suggest that this may be circuit-specific, and these aspects will be examined by us in future studies.

On the other hand, a study evaluating the enzyme activity of another peptidase, namely Angiotensin I-converting enzyme (ACE), which shares common substrates with NDEL1 such as neurotensin (NT), also showed region-specific alterations in the rat brain after chronic treatment with different neuroleptic drugs^39,56^. This specificity may be directly related to the neuropeptide system^57^, or it may possibly be a response to the demonstrated increase in neuronal activity in the nucleus accumbens due to chronic treatment with neuroleptics such as haloperidol^58^. Moreover, antipsychotic-induced dopamine supersensitivity, which decreased serotonin 5-HT2A receptor density in the nucleus accumbens (whereas it increased in other brain regions such as the caudate putamen) and enhanced ability of 5-HT2/5-HT2A receptors to modulate dopamine-dependent behaviors, could also be linked to observed changes in 5-HT2A receptor density in the prefrontal cortex and striatum^59^. More interestingly, NT in the nucleus accumbens was found to reverse this dopamine supersensitivity caused by antipsychotic treatment^60^. Therefore, besides confirming that heterogeneous responses in the brain would be expected in response to chronic treatment with antipsychotics, this work also suggests a possible mechanism involving NT and serotonin pathway to explain the differential response of NWR and SHR strains following antipsychotic treatment.

Taken together, these data reinforce our hypothesis that decreases in NDEL1 activity observed exclusively in the animal model for SCZ following the antipsychotic exposure may reflect progressive neurobiological changes in the brain toward a SCZ-like phenotype, which may not be completely stopped by the antipsychotic medication regardless of the amelioration of SCZ symptoms, as also suggested in our recent work with antipsychotic-naïve FEP SCZ patients^31^.

In contrast to the significant increase of NDEL1 enzyme activity with animal aging only in NWR (Supplementary Fig. 1), it is worthwhile to note that after long-term treatment with (typical or atypical) antipsychotics, NDEL1 activity was decreased mainly in the SHR animals, with no significant changes in blood or in different brain regions of NWR, with an exception for the prefrontal cortex and nucleus accumbens. At this point, we hypothesize that NDEL1 activity may not be modulated by antipsychotics in the healthy controls (HCs), based on the observation in control NWR strain, possibly because their therapeutic effects may involve the restoration of deficits, and are only effective in altered conditions, such as those observed in the animal model for SCZ. Therefore, although we have suggested that the lower NDEL1 enzyme activity observed in FEP patients compared to HCs may be more likely reflecting a possible acute response to the illness induced alterations, the effects of the antipsychotics on NDEL1 activity were also recognized^31^. In addition, the pharmacological effect of the therapy with antipsychotics may be effective for the amelioration of a specific set of symptoms but not necessarily for all other impairments, such as the well-known prefrontal myelination and white/grey matter losses^61,62^. These findings are in line with the present study employing this animal model for SCZ to evaluate the effects of typical and atypical antipsychotics treatment on NDEL1 enzyme activity.

In addition, the systolic blood pressure (BP) and heart rate of these animals monitored before and after treatment with these typical and atypical antipsychotics showed no significant difference in these parameters in either rat strains^39^, which may preclude the possible influence of cardiovascular parameters in NDEL1 enzyme activity, as measured here, or in animal behaviors, which were previously evaluated by others^36–38^.

Moreover, potential effects of stimulated dopamine release on NDEL1 enzyme activity was also evaluated by single injections of amphetamine (AMPH) or lisdexanfetamine (LDX) in the NWR animals, which showed significant increases in NDEL1 activity (Fig. 4), regardless of the BP changes observed only with AMPH but not with the prodrug LDX (Table 3). Our hypothesis of a possible modulation of NDEL1 activity secondary to dopamine homeostasis as an underlying mechanism of action mediating AMPH-induced NDEL1 activity will be most probably confirmed after conducting more specific studies, which might be addressed in the near future.

We recognize as limitations of this work, the potential genetic differences between the rat strains studied here, although this fact may not influence the main objective, which was to show the significant decrease in NDEL1 activity following a chronic treatment with typical or atypical antipsychotics only in the strain presenting SCZ-like phenotypes. Also, we did not investigate the influence of sex in the present work, since we have used only male rats. In addition, the control normal rat strain (showing no SCZ-like phenotypes) allowed to demonstrate significant increases of NDEL1 enzyme activity after acute administration of psychostimulants, under conditions in which SCZ-like phenotype could be induced in these animals^36–38^. Interestingly, the observed changes in NDEL1 enzyme activity was in line with those demonstrated in individuals with FEP after 2-months and one-year follow-up studies under treatment with risperidone^31^. Additional studies using a different animal model were also recently performed by us to corroborate the data presented here, and the NDEL1 activity associated with changes with brain malformation could be demonstrated^63^. Now, to better understand the mechanism(s) underlying the modulation of NDEL1 activity secondary to dopamine homeostasis, we may also evaluate the brain formation of SHR animals in utero to enable the assessment of therapeutic interventions that could potentially decrease the risk of developing SCZ-like phenotypes or, at least, the rate of illness onset and progression, which seems to be dissociated from the amelioration of symptoms determined by dopaminergic antagonism.

Lastly, NDEL1 activity clearly increases with aging, but this increase was significantly smaller in the SHR animals compared to the normal control animals (namely NWR) (Supplementary Fig. 1). This activity in SHR was also significantly decreased by the treatment with typical or atypical antipsychotics, which is in line with the age-dependent differences in NDEL1 enzyme activity observed by us in FEP and chronically treated SCZ patients^31^.

## Conclusion

There are three main conclusions to be drawn from the present study, which are: (a) NDEL1 activity in blood mirrors the levels of enzyme activity for most of the brain regions investigated, even before or after treatment with typical or atypical antipsychotics; (b) blood levels of NDEL1 activity is also modulated by the administration of other dopaminergic modulators, such as the psychostimulants, although antipsychotics can modulate NDEL1 activity only in the animal model for SCZ; and lastly, (c) these drugs can also affect NDEL1 enzyme activity in the blood, regardless of the blood pressure changes. Thus, NDEL1 enzyme activity may represent a valuable tool to investigate SCZ neurobiology and also the effects of drugs which modulate dopamine homeostasis. Further investigation of the pathways underlying SCZ, including the potential role of increased dopamine release for SCZ susceptibility and subsequent modulation of NDEL1 enzyme activity, is also being considered. Present data provides new insights in the role of NDEL1 and its possible interactions with neurotransmitter systems such as the dopaminergic pathway, and the potential liability of this pathway involving neuropeptides and neuropeptidases on susceptibility for severe mental disorders.

## Supplementary information

Supplementary Information

## Abbreviations

SCZ: Schizophrenia
SHR: Spontaneously hypertensive rats
NWR: Normotensive Wistar rats
NDEL1: Nuclear distribution element-like 1
BK: Bradykinin
NT: Neurotensin
HCs: Healthy controls
FEP: First-episode of psychosis
Cloz: Clozapine
Halo: Haloperidol
*ip*: Intraperitoneal
Pf-Ctx: Pre-frontal cortex
Hippo: Hippocampus
Stri: Striatum
nAcc: Nucleus accumbens
FRET: Fluorescence resonance energy transfer
AUF: Arbitrary units of fluorescence
AMPH: Amphetamine
LDX: Lisdexanfetamine
BP: Blood pressure
HR: Heart rate
