# Supplementary material for: Evaluation of NDEL1 oligopeptidase activity in blood and brain in an animal model of schizophrenia: effects of psychostimulants and antipsychotics
Source: Sci Rep. 2020 Oct 28;10:18513. doi: 10.1038/s41598-020-75616-2 (PMC7595172; doi:10.1038/s41598-020-75616-2)
Supplement: Supplementary file 1 — Supplementary Information [file 41598_2020_75616_MOESM1_ESM.docx]

**Supplemental material**

**Evaluation of NDEL1 oligopeptidase activity in blood and brain in an animal model of schizophrenia: effects of psychostimulants and antipsychotics**

João V. Nani^1,2,3^, Richard S. Lee^4^, Camila M. Yonamine^1,3^, Osvaldo A. Sant'Anna^5^, Maria A. Juliano^6^, Ary Gadelha^2^, Jair J. Mari^2^, Mirian A. F. Hayashi^1,3,^*

^1^Department of Pharmacology, Escola Paulista de Medicina (EPM), Universidade Federal de São Paulo (UNIFESP), Brazil; **^2^**Department of Psychiatry, Escola Paulista de Medicina (EPM), Universidade Federal de São Paulo (UNIFESP), Brazil;^3^ Laboratory of Immunochemistry, Instituto Butantan, São Paulo, Brazil; ^4^Department of Biophysics, Escola Paulista de Medicina (EPM), Universidade Federal de São Paulo (UNIFESP), Brazil; ^5^National Institute for Translational Medicine (INCT-TM, CNPq/FAPESP/CAPES) , Ribeirão Preto , Brazil.

*Corresponding author:

Prof. Mirian A. F. Hayashi, *Ph.D.*

Departamento de Farmacologia, Escola Paulista de Medicina (EPM), Universidade Federal de São Paulo (UNIFESP), Rua 3 de maio 100, Ed. INFAR, 3^rd^ floor, CEP 04044-020, Tel +55-11-5576 4447/FAX +55-11-5576 4499, São Paulo, Brazil;

e-mail: [mhayashi@unifesp.br](mailto:mhayashi@unifesp.br) or [mirianhayashi@yahoo.com](mailto:mirianhayashi@yahoo.com)

**Abbreviations**: SCZ, schizophrenia; SHR, spontaneously hypertensive rats; NWR, normotensive Wistar rats; FRET, fluorescence resonance energy transfer; AUF, arbitrary units of fluorescence.

**Short Title:** NDEL1 activity in animal model for schizophrenia.

**NDEL1 enzyme activity in the serum of drug-naïve normotensive Wistar rats (NWRs) and spontaneously hypertensive rats (SHRs).** Male Drug-naïve, normal Wistar **(**NWR) and spontaneously hypertensive (SHRs) rat strains (3 and 5 months old) were used to perform this experiment. The blood of rats was collected after euthanasia, and serum was used for the measurements of the NDEL1 enzyme activity. The NDEL1 enzyme activity was measured as described in the Methods section of manuscript.

The mean values for NDEL1 enzyme activity in the serum of 5 month-old Drug-naïve normotensive Wistar rats (NWR) and spontaneously hypertensive rats (SHRs) were determined as 4.2 ± 0.7 and 7.1 ± 1.3 nM/min (mean ± SD), respectively, showing a significant higher NDEL1 activity in the blood of SHR compared to NWR animals, as also observed at young adult age (*i.e.* 3 months old) (two-way ANOVA (F (1, 15) = 40.03, *p* = 0.0001) (Figure 1). We also observed increases of the NDEL1 enzyme activity with increasing age in both strains, which was statistically significant only for NWR strain (post hoc Tukey test, *p =* 0.0067), while the numerical increase observed in SHR animals was not statistically different (post hoc Tukey test, *p =* 0.1668), at least for the 2-month period (Supplemental Figure 1).


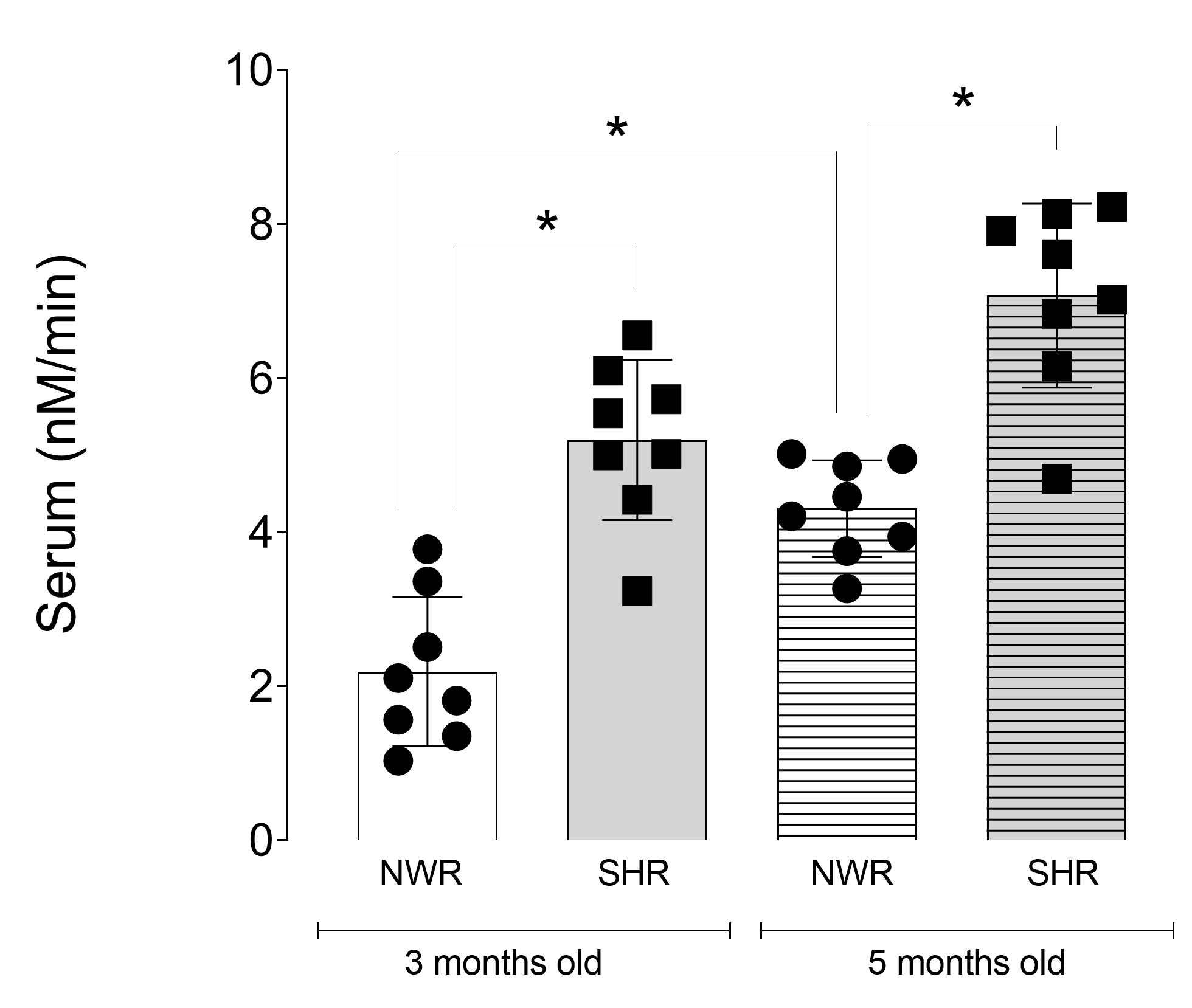


**Supplemental Figure 1. NDEL1 enzyme activity in the serum of normotensive Wistar rats (NWRs) and spontaneously hypertensive rats (SHRs).** The blood of rats was collected after euthanasia and serum was used for the measurements of the NDEL1 enzyme activity (N = 8 per group, *p < 0.05, two-way ANOVA, post-hoc test Tukey for multiple comparisons).
